# Anesthesia and Analgesia Methods in Primary Total Hip Arthroplasty and Primary Total Knee Arthroplasty—A Survey of Nordic Anesthesiologists

**DOI:** 10.1111/aas.70091

**Published:** 2025-07-17

**Authors:** Maarit Rantakokko, Marcus Teräs, Pekka Tarkkila, Andreas Wiklund, Martin Ingi Sigurðsson, Gjermund Galleberg, Sine Wichmann, Panu Uusalo

**Affiliations:** ^1^ Department of Anaesthesiology and Intensive Care University of Turku Turku Finland; ^2^ Division of Perioperative Services Intensive Care and Pain Medicine, Turku University Hospital Turku Finland; ^3^ Department of Anaesthesiology and Intensive Care University of Helsinki Helsinki Finland; ^4^ Capio Artro Clinic Stockholm Sweden; ^5^ Department of Anaesthesiology and Critical Care Medicine Landspitali – The National University Hospital of Iceland, Reykjavik Iceland, and Faculty of Medicine, University of Iceland Reykjavik Iceland; ^6^ Department of Anaesthesia and Intensive Care Haukeland University Hospital Bergen Norway; ^7^ Department of Anaesthesiology and Intensive Care Copenhagen University Hospital – North Zealand Hillerod Denmark

**Keywords:** day case surgery, multimodal anesthesia, total hip arthroplasty, total knee arthroplasty

## Abstract

**Background:**

The objective of this survey was to assess the current practices of analgesia and anesthesia for patients undergoing primary hip (THA) and knee (TKA) total joint arthroplasty in the Nordic countries. Additionally, we aimed to explore the differences in anesthesia and analgesia techniques, prevalence of day case surgery procedures, criteria for patient selection, and the challenges associated with patient discharge.

**Methods:**

An online survey was created and distributed to all anesthesiologists of Nordic orthopedic surgical units conducting over 100 arthroplasties a year according to national arthroplasty registries.

**Results:**

Out of 298 survey responses, 94.3% reported following a standard operation procedure (SOP). Preoperative medication was used by 65.1% for THA and 63.1% for TKA patients. Intraoperative corticosteroids were administered by 79.2% for THA and 81.7% for TKA patients. Spinal anesthesia was used for THA (95.6%) and TKA (92.3%), with bupivacaine preferred for spinal anesthesia in THA (83.9%) and TKA (88.4%). Local infiltration analgesia (LIA) was used for 37.6% of THA and 64.4% of TKA patients. Peripheral nerve blocks were administered by 8.1% for THA and 40.9% for TKA patients. Postoperative pain medications included opioids (96.0%), paracetamol (93.0%), NSAIDs including COX‐2 inhibitors (81.9%), and gabapentinoids (8.4%). Antiemetics were used by 43.7%. Nearly half of respondents (49.7%) from 61 hospitals reported performing primary THA and TKA as day‐case procedures, but less than 25% of patients had day surgery. Delayed discharge reasons included intense pain, motor weakness, and postoperative nausea and vomiting (PONV).

**Conclusions:**

There is general agreement in the Nordic countries on preoperative medication, anesthesia techniques, and multimodal pain management, though variability exists in the use of peripheral nerve blocks and LIA. Day‐case TJAs are common, especially in Denmark. Pain, motor weakness, and PONV are the main barriers to same‐day discharge. The survey suggests that better management of PONV with consistent use of antiemetics could improve recovery and reduce discharge delays.

This report describes survey results about anesthesia management preferences for anesthesia management for adult hip and knee arthroplasty cases in Nordic countries. The findings demonstrate practice focus on early recovery where possible.

## 1 Introduction

The aging population is driving an increasing demand for total joint arthroplasty (TJA) procedures, which offer patients enhanced mobility and pain relief, resulting in improved quality of life [1]. This growing need for TJAs is occurring in the context of reduced hospital capacity and the implementation of austerity measures, necessitating more efficient healthcare delivery.

Perioperative care is crucial in the recovery process following TJAs, with anesthesia and analgesia methods significantly impacting patient outcomes. Despite extensive research into optimal TJA anesthetic techniques, ongoing debates persist regarding the most effective approaches [2, 3, 4].

In response to these challenges, there is a rising trend of performing TJAs as day surgeries. This approach may offer cost savings, faster recovery, improved patient satisfaction, and reduced workload for surgical wards [5]. However, concerns regarding feasibility and patient safety remain, underscoring the need for continued refinement of care strategies.

The aim of this study was to survey and evaluate the current anesthesia and analgesia practices of patients undergoing total hip arthroplasty (THA) and total knee arthroplasty (TKA) in Nordic hospitals. Our objectives were to evaluate the extent to which the methods currently employed are aligned with established guidelines and promote an in‐depth discussion on the methods currently in use.

## 2 Materials and Methods

### 2.1 Study Design

This study was an online cross‐sectional survey. Participants were anesthesiologists with different experience levels, working in a department of anesthesiology and intensive care in Denmark, Finland, Iceland, Norway, and Sweden. We aimed to obtain all Nordic hospitals that perform more than 100 TJAs annually. These hospitals were identified from the Nordic Arthroplasty Registries for the year 2023, with 37 hospitals in Denmark, 29 in Finland, 38 in Norway, and 64 in Sweden. The Icelandic Arthroplasty Register was not available for our use. A national coordinator from each country collaborated on the study and distributed the questionnaire to hospitals that fulfilled the criteria.

### 2.2 Survey Development

The survey was created and processed using Research Electronic Data Capture (REDcap). The survey and final manuscript adhere to the CROSS checklist.

The survey was based on a formative model and written in English. The survey items were developed based on published literature, incorporating recommendations such as using unbiased vocabulary, employing response anchors that emphasize the construct being measured, and selecting relevant response options for each item. The questions included a mix of close‐ended formats, dichotomous answers, and various Likert‐type response anchors.

The survey consisted of five parts:

1. Demographic data (Q1‐Q5)
2. Anesthesia and analgesia methods in primary THA (Q6‐Q33)
3. Anesthesia and analgesia methods in primary TKA (34‐Q61)
4. Anesthesia and analgesia practice patterns in primary THA and TKA (Q62‐Q76)
5. Primary THA and TKA as day surgery (Q77‐Q86)

The detailed questionnaire is provided as Supplemental file 1.

### 2.3 Survey Administration

Data was obtained and kept through an online survey using REDCap. Participants were invited via email, in agreement with the chief physician of the relevant departments. An email reminder was sent out 2 weeks following the initial invitation and both surveys were open for 40 days. The survey was conducted in Finland between August 14, 2024 and September 23, 2024, and in the other Nordic countries between November 11, 2024 and December 21, 2024.

### 2.4 Statistical Analysis

Data analysis was performed with JMP Pro 17 (SAS, Cary, NC, 2022). Data was mainly analyzed and summarized using a non‐parametric descriptive approach. A Kruskal–Wallis test was used to examine relevant relationships between countries, hospital types, and work experience of anesthesiologists. All statistics were considered significant at a *p* < 0.05. Incomplete responses with missing data were excluded from the analysis.

### 2.5 Ethics

A notification was sent to the regional Scientific Ethics Committee in the Region of South‐West Finland. As no patient data is involved in this descriptive study, with no direct effect on patient management, no ethical committee approval was required. The findings of this study reflect anesthesiologists' perceptions of patient courses and treatments, rather than actual clinical outcomes.

## 3 Results

**FIGURE 1:**
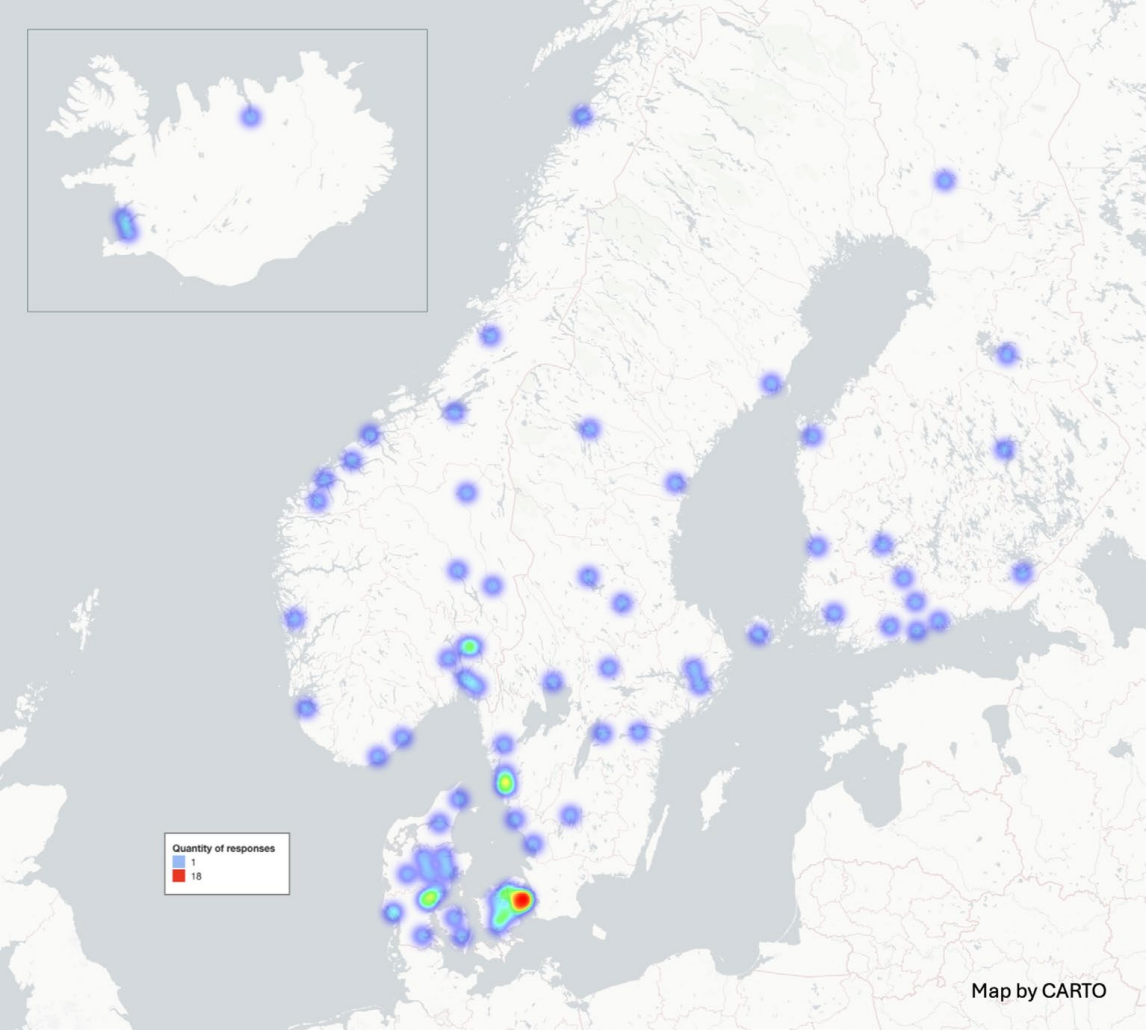
Heatmap representing the distribution and location of the survey respondents.

Demographic data regarding the respondents is summarized in Table 1. The survey was distributed to 168 hospitals, and a total of 298 anesthesiologists from 105 different hospitals participated in the study. Of these, 180 (60.4%) were from Denmark, sourced from 35 different hospitals or units, 37 (12.4%) from Finland from 19 different hospitals, 36 (12.1%) from Norway from 24 different hospitals, 31 (10.4%) from Sweden from 24 different hospitals, and 14 (4.7%) from Iceland from 3 different hospitals (Figure 1). Most respondents, 203 (68.1%), were consultants with work experience of over 5 years. Additionally, 120 (40.3%) treated more than 80 arthroplasty cases annually. The majority of respondents, 281 (94.3%), followed a SOP at their work.

**TABLE 1:** Demographics of the survey respondents.

Respondents by country—*n* (%)	
Finland	37 (12.4)
Iceland	14 (4.7)
Norway	36 (12.1)
Sweden	31 (10.4)
Denmark	180 (60.4)
Workplace of respondents—*n* (%)	
Private hospital	21 (7.0)
Regional hospital	158 (53.0)
University hospital	119 (39.9)
Work experience of respondents—*n* (%)	
Resident	50 (16.8)
Consultant 0–5 years	45 (15.1)
Consultant > 5 years	203 (68.1)
Annual THA/TKA case volume per anesthesiologist—*n* (%)	
0–40	103 (34.6)
40–80	75 (25.2)
> 80	120 (40.3)
Standard operation procedure (SOP)	
Follow SOP	281 (94.3)
Own preference	17 (5.7)

*Note:* Data are given as amount (*n*) and percentage.

### 3.1 Preoperative Medication and Anesthesia of Patients Undergoing THA or TKA

Preoperative medication was administered by 194 (65.1%) of respondents for THA and 122 (63.1%) for TKA, with paracetamol being the most commonly used analgesic in both procedures—122 (63.1%) for THA and 180 (95.7%) for TKA, followed by NSAIDs used by 85 (44.0%) and 125 (66.5%), respectively. The highest rates of preoperative medication use were reported in Norway, where all 36 respondents administered them for both THA and TKA, and in Sweden, where 29 (93.5%) administered them for THA and 27 (87.1%) for TKA. Only three respondents reported using benzodiazepine premedication for both THA and TKA.

Intraoperative corticosteroids were administered by the majority of respondents for both THA 236 (79.2%) and TKA 243 (81.7%), with dexamethasone being the most frequently utilized agent.

Spinal anesthesia was the most commonly chosen technique for both THA and TKA, used by 285 (95.6%) and 275 (92.3%) respondents, respectively. General anesthesia was chosen as the primary anesthesia method for THA and TKA by 8 (2.7%) and 2 (6.5%), respectively. Hypobaric (plain) bupivacaine was used as the local anesthetic for THA by 250 (83.9%) respondents. For TKA, 250 (84.1%) respondents reported using bupivacaine. Both hyperbaric (48.4%) and hypobaric (plain) (51.6%) local anesthetics were used almost equally in TKA. Ropivacaine was employed for THA and TKA by 34 (11.4%) and 32 (10.7%) respondents, respectively. For TKA, prilocaine was also used by 5 (1.7%) of respondents. The lowest median dose of intrathecal bupivacaine was reported by respondents from Denmark (THA median dose 12.0 mg, 95% CI: 11.0–12.5 mg; TKA median dose 11.6 mg, 95% CI: 10.0–12.5 mg), by private hospitals (THA median dose 8.9 mg, 95% CI: 6.5–11.0 mg; TKA median dose 10.0, 95% CI: 7.5–11.8 mg), and by anesthesia residents (THA median dose 11.0 mg, 95% CI: 10.0–12.5 mg; TKA median dose 10.0 mg, 95% CI: 10.0–12.5 mg). The doses of bupivacaine and ropivacaine, as well as the proportions of local infiltration analgesia (LIA) and regional blocks, are detailed in Tables 2, 3, 4.

**TABLE 2:** Regional anesthesia for THA and TKA by country.

(a) THA						
	SWE	NOR	DEN	ISL	FIN	*p*
Intrathecal bupivacaine dose (mg)	12.5 (11.0–15.0)	13.5 (11.5–15.0)	12.0 (10.0–12.5)	13.4 (12.4–15.6)	12.0 (10.0–13.5)	*< 0.001*
Intrathecal ropivacaine dose (mg)	N/A	N/A	15.0 (12.5–15.0)	N/A	15.0 (15.0–19.0)	0.07
LIA, *n* (%)	13 (41.9)	9 (25.0)	65 (36.1)	4 (28.6)	21 (56.8)	0.06
Regional block, *n* (%)	4 (12.9)	2 (5.6)	16 (8.9)	1 (7.1)	1 (2.7)	0.52
Femoral block	1	1	8	0	0	
PENG block	2	1	4	1	1	
Fascia iliaca block	1	0	1	0	0	
Other	0	0	3	0	0	

(b) TKA						
	SWE	NOR	DEN	ISL	FIN	*p*
Intrathecal bupivacaine dose (mg)	12.5 (10.0–14.1)	12.5 (11.0–14.0)	11.6 (10.0–12.6)	12.5 (11.6–15.0)	12.0 (10.0–12.5)	*0.004*
Intrathecal ropivacaine dose (mg)	15.0 (15.0–15.0)	12.5 (10.0–15.0)	13.5 (12.5–15.0)	N/A	13.0 (13.0–13.0)	0.731
LIA, *n* (%)	27 (87.1)	26 (72.2)	92 (51.1)	10 (71.4)	37 (100)	< *0.001*
Regional block, *n* (%)	3 (9.7)	18 (50.0)	81 (45.0)	5 (35.7)	15 (40.5)	*0.003*
Femoral block	0	0	2	0	0	
Adductor block	2	9	58	4	14	
iPACK	1	4	10	1	1	
Other	0	5	12	0	0	

*Note:* Data are given as median and interquartile range or as amount (*n*) and percentage. *p* < 0.05 in italics.

Abbreviations: DEN, Denmark; FIN, Finland; iPACK, infiltration of local anesthetic between the popliteal artery and capsule of the knee; ISL, Iceland; NOR, Norway; SWE, Sweden; THA, total hip arthroplasty; TKA, total knee arthroplasty.

**TABLE 3:** Regional anesthesia for THA and TKA by hospital.

(a) THA				
	UNIV	REG	PRIV	*p*
Intrathecal bupivacaine dose (mg)	12.0 (10.0–13.3)	12.8 (12.0–15.0)	8.9 (6.3–11.0)	*< 0.001*
Intrathecal ropivacaine dose (mg)	15.0 (15.0–20.0)	15.0 (13.0–15.0)	N/A	0.07
LIA, *n* (%)	54 (45.4)	49 (31.0)	9 (42.9)	*0.04*
Regional block, *n* (%)	7 (5.9)	16 (10.1)	1 (4.8)	0.37
Femoral block	1	9	0	
PENG block	3	5	1	
Fascia iliaca block	1	1	0	
Other	2	1	0	

(b) TKA				
	UNIV	REG	PRIV	*p*
Intrathecal bupivacaine dose (mg)	10.0 (10.0–12.5)	12.5 (11.5–14.0)	10.0 (7.1–10.9)	*< 0.001*
Intrathecal ropivacaine dose (mg)	15.0 (13.0–15.0)	14.0 (12.5–15.0)	N/A	0.30
LIA, *n* (%)	91 (76.5)	82 (51.9)	19 (90.5)	*< 0.001*
Regional block, *n* (%)	56 (47.0)	60 (38.0)	6 (28.6)	0.22
Femoral block	0	2	0	
Adductor block	47	34	6	
iPACK	5	12	0	
Other	4	13	0	

*Note:* Data are given as median and interquartile range or as amount (*n*) and percentage. *p* < 0.05 in italics.

Abbreviations: iPACK, infiltration of local anesthetic between the popliteal artery and capsule of the knee; LIA, local infiltration analgesia; PRIV, private hospital; REG, regional hospital; THA, total hip arthroplasty; TKA, total knee arthroplasty; UNIV, university hospital.

**TABLE 4:** Regional anesthesia for THA and TKA by work experience.

(a) THA				
	Resident	0–5 years consultant	> 5 years consultant	*p*
Intrathecal bupivacaine dose (mg)	11.0 (10.0–12.5)	12.5 (10.0–12.6)	12.5 (10–15)	*0.019*
Intrathecal ropivacaine dose (mg)	15.0 (15.0–15.0)	13.0 (12.5–13.0)	15.0 (3.8–15.0)	0.26
LIA, *n* (%)				0.17
Regional block, *n* (%)	8 (16)	4 (8.9)	12 (5.9)	0.09
Femoral block	6	1	3	
PENG block	1	3	5	
Fascia iliaca block	1	0	1	
Other	0	0	3	

(b) TKA				
	Resident	0–5 years consultant	> 5 years consultant	*p*
Intrathecal bupivacaine dose (mg)	10.0 (10.0–12.5)	11.0 (10.0–12.5)	12.5 (10.0–13.8)	*0.001*
Intrathecal ropivacaine dose (mg)	11.8 (10.0–15.0)	12.5 (12.5–15.0)	14.0 (12.5–15.0)	0.32
LIA, *n* (%)	21 (43.8)	29 (65.9)	142 (71.7)	*0.002*
Regional block, *n* (%)	22 (44.0)	17 (37.8)	83 (40.9)	0.78
Femoral block	1	0	1	
Adductor block	17	9	61	
iPACK	3	6	8	
Other	1	2	14	

*Note:* Data are given as median and interquartile range or as amount (*n*) and percentage. *p* < 0.05 in italics.

Abbreviations: iPACK, infiltration of local anesthetic between the popliteal artery and capsule of the knee; LIA, local infiltration analgesia; THA, total hip arthroplasty; TKA, total knee arthroplasty.

A total of 24 (8.1%) respondents regularly used a peripheral nerve block for THA; of these, 10 (41.7%) used a femoral block without a catheter, 9 (37.5%) used a pericapsular nerve group (PENG) block, and 2 (8.3%) used a fascia iliaca block. No significant differences were found in the use of nerve blocks based on country, hospital type, or work experience.

For TKA, 122 (40.9%) respondents used a peripheral nerve block routinely, with the highest use in Norway (*p*=0.003). According to 259 (86.9%) respondents, anesthetic technique did not affect the decision to use a nerve block. Among those utilizing a peripheral nerve block, 87 (71.3%) applied the adductor canal block without a catheter, while 17 (13.9%) used infiltration of local anesthetic between the popliteal artery and capsule of the knee (IPACK), 14 (11.5%) used a popliteal block, and 2 (1.6%) used a femoral block.

A majority of respondents, 277 (93.8%) for THA and 280 (92.7%) for TKA, administered sedatives in conjunction with spinal anesthesia. Propofol was used by 256 (92.7%) for THA and 275 (98.2%) for TKA, followed by midazolam, used by 53 (19.3%) for THA and 57 (20.4%) for TKA.

LIA administered by the surgeon was reported to be used by 112 (37.6%) respondents for THA, with highest use in university hospitals (*p* = 0.04) but with no significant differences across countries or work experiences. Only 7 (16%) of THA patients receiving LIA had an additional peripheral nerve block. For TKA, 192 (64.4%) respondents used LIA, with the highest use in Finland (*p* < 0.001), in private hospitals (*p* < 0.001) and among consultants with work experience of over 5 years (*p* = 0.002). Of TKA patients receiving LIA, 80 (41.7%) were administered a peripheral nerve block. The most common medications utilized in LIA included ropivacaine, used by 127 (66.1%) respondents, epinephrine, used by 84 (43.8%) respondents, NSAID, used by 60 (31.2%) respondents, and bupivacaine, used by 50 (26.2%) respondents.

### 3.2 Postoperative Care of Patients Undergoing THA and TKA

Nearly all respondents, 283 (95.0%), employed multimodal postoperative pain management in primary THA and TKA. In addition to LIA and peripheral nerve blocks, the most frequently used analgesics were opioids, used by 286 (96.0%) respondents, paracetamol, used by 277 (93.0%), NSAIDs including COX‐2 inhibitors, used by 244 (81.9%), and gabapentinoids, used by 25 (8.4%). Clonidine was administered postoperatively by 12 (4.0%) and esketamine by 11 (3.7%) respondents.

Not including corticosteroids, routine antiemetic prophylaxis was utilized by 129 (43.7%) respondents. 96 (32.2%) respondents reported routinely using one additional antiemetic, 23 (7.7%) using two, and 3 (1.0%) using three additional antiemetics. In Sweden, the median (IQR) number of additional antiemetics was 1 (0–1), whereas in other countries it was 0 (0–1) (*p* = 0.002). There was no difference in the number of additional antiemetics between workplaces or levels of work experience. Among those who prescribed antiemetic prophylaxis, the most commonly used antiemetics were 5‐hydroxytryptamine (5HT3) antagonists, prescribed by 119 (92.2%) respondents, droperidol, used by 21 (16.3%), and NK1 inhibitors, used by 6 (4.7%) respondents.

### 3.3 Primary THA and TKA as Day Surgery

A total of 148 respondents (49.7%) reported working in units offering THA and TKA as day surgery, including 21 (7.0%) from 11 hospitals in Finland, 102 (34.2%) from 32 hospitals in Denmark, 14 (4.7%) from 11 hospitals in Sweden, 9 (3.0%) from 6 hospitals in Norway, and 2 (0.7%) from 1 hospital in Iceland. In the units offering day surgery, these procedures typically accounted for < 25% of all primary THA and TKA operations. In most cases, 113 (89.9%), the anesthesia and analgesia protocols employed were consistent with those used for in‐hospital patients. In patient selection, 77 (52.0%), 74 (50.0%), and 60 (40.5%) respondents indicated that age above 75 years, a BMI greater than 35 kg/m^2^, or obstructive sleep apnea requiring CPAP treatment, respectively, did not alone preclude patients from undergoing day surgery.

The primary reasons for delayed discharge in planned day surgery were perceived as prolonged or intense pain, reported by 104 (70.3%) respondents, motor weakness, reported by 61 (41.2%), and PONV, reported by 40 (27.0%). Follow‐up calls were made in 54 (36.5%) cases.

## 4 Discussion

The results of this survey provide insights into the current trends in anesthesia and analgesia practices among Nordic anesthetists in TJA surgeries. The survey was specifically distributed to orthopedic units, and the broad range of responses received from all participating countries supports the reliability of the findings. Several areas of consensus, in line with current recommendations, are observed in preoperative medication management, anesthesia techniques, and multimodal pain management strategies across the region [6, 7, 8]. However, variability exists especially in the use of peripheral nerve blocks and LIA. The majority of respondents also employed a SOP, reflecting highly standardized treatment protocols and accounting for the consistent responses observed.

Multimodal analgesia, which combines multiple analgesic techniques, is considered the cornerstone of modern pain management for THA and TKA. Commonly used components include paracetamol, NSAIDs, and rescue opioids [3, 9, 10]. The vast majority of respondents reported using multiple analgesics both pre‐ and postoperatively, of which a small number also reported using gabapentinoids, clonidine, and in some cases also esketamine.

Spinal anesthesia was the prevailing anesthesia method for both THA (95.6%) and TKA (92.3%). While current guidelines consider both general and spinal anesthesia to be viable options [6], the preference for spinal anesthesia may be more rooted in tradition than in clear, evidence‐based advantages under modern anesthetic practice. Smaller doses of local anesthetics were most frequently reported in Denmark and in private clinics, likely reflecting contextual factors such as Denmark's extensive experience with fast‐track surgery and the refined care pathways that accompany it. In private clinics, anesthesiologists may prefer lower doses of spinal anesthetics due to the controlled environment, predictable surgical durations, and efficient care pathways, facilitating faster recovery and enabling same‐day discharge. Notably, anesthesia residents were among those administering the lowest doses, possibly due to stricter adherence to standardized protocols or the fact that most of the residents 47 (94.0%) were trained in Denmark.

Excessive motor blockade was the second most commonly cited reason for delayed discharge in day surgery patients, reported by 41.2% of respondents, indicating that further reductions in spinal anesthetic dosage or the use of shorter‐acting agents could enhance postoperative outcomes. Although 14.9% of respondents reported using general rather than spinal anesthesia for day‐case TJAs, this approach presents challenges in managing immediate postoperative pain and PONV. Anesthesiologists also identified pain, motor weakness—often associated with prolonged spinal or peripheral nerve blocks—and PONV as primary factors contributing to delayed discharge, a finding that aligns with existing literature [11]. The survey indicates an interest in the use of shorter‐acting anesthetics, with ropivacaine commonly used for both THA and TKA, and prilocaine primarily for TKA. Despite these developments, many respondents indicated that anesthesia techniques typically used for inpatient procedures continue to be applied in outpatient settings.

There was considerable variation in the use of LIA and peripheral nerve blocks across the Nordic countries. The use of LIA was reported more frequently in Finland for both THA and TKA, with the difference being statistically significant for TKA. The ERAS protocol recommends the use of LIA for TKA as part of a multimodal, opioid‐sparing analgesic strategy but does not recommend it for THA [6]. Conversely, the PROSPECT guideline suggests that either a single‐shot fascia iliaca block or LIA may be appropriate for THA [3]. Among TKA patients, the combined use of LIA and a peripheral nerve block was most commonly reported in Finland 15 (40.5%) and in university hospitals 47 (39.5%). No clear explanation for these differences can be drawn from the current data. Clinical guidelines are not fully aligned, and existing research findings are, to some extent, contradictory. The use of peripheral nerve blocks in combination with LIA remains a topic of debate, as LIA alone has been shown to provide effective analgesia without inducing motor blockade [12]. However, in situations where patients experience substantial postoperative pain and where early mobilization and prompt discharge are prioritized, the use of peripheral nerve blocks may constitute a logical and efficacious approach to analgesia. The more frequent use of regional anesthesia techniques in university hospitals may be attributed to their larger orthopedic departments and greater availability of specialized expertise and resources.

Based on the survey findings, there appears to be room for improvement in the overall use of antiemetics, as 27% of respondents reported PONV as the third most common reason for delayed discharge following planned day surgery for TJA. Most respondents routinely administered perioperative corticosteroids, particularly dexamethasone, to patients undergoing TJA. Additionally, 32.2% reported using one supplementary antiemetic. This practice was slightly more common among Swedish respondents and aligns with current guidelines recommended for patients with one to two PONV risk factors [13]. Enhanced recovery protocols further advocate this approach even without identifiable risk factors. However, the survey did not assess whether PONV risk stratification was performed preoperatively, which is strongly recommended to guide appropriate prophylaxis [13].

Day‐case surgery for TJA is becoming more common for well‐selected patients, offering cost savings and comparable risks of complications and re‐operations to traditional in‐hospital procedures [14]. In the survey, 32 units (91.4%) in Denmark offer outpatient TJA, compared to 11 (57.9%) in Finland, 6 (25%) in Norway, and 11 (45.8%) in Sweden. These findings demonstrate Denmark's leading role in the development and implementation of fast‐track TJA protocols across the Nordic region, likely reflecting its earlier and more systematic adoption of these practices [14, 15, 16, 17, 18, 19, 20, 21]. Despite the increasing demand for day‐case procedures, a recent study found that only 41% of patients expressed interest in day‐case arthroplasty, underscoring the need for improved preoperative education and greater patient involvement in treatment decision‐making [14]. One reason for the lower utilization of day‐case surgeries could be the geographical challenges and limited access to specialized centers in certain countries or regions. Additionally, day‐case surgery might not always align with the financial interests of the healthcare providers.

Postoperative follow‐up for day‐case patients was provided to a limited degree (36.5%). Increasing the proportion of follow‐up care would help in understanding the issues patients face in the home setting, as well as enhance patient safety and motivation for day‐surgery procedures [22].

### 4.1 Limitations and Strengths

This study has several limitations. Firstly, the findings reflect anesthesiologists' perceptions of patient courses and treatments, rather than objective clinical outcomes. Secondly, the response rate to the survey could not be determined; therefore, the potential impact of non‐response bias cannot be assessed. We are also aware that some of the surveys may have ended up in the recipients' email spam folders. Thirdly, a disproportionately large number of respondents were Danish, which reduces the generalizability of the results to the Nordic countries as a whole. Fourthly, the survey did not distinguish between the treatment of postoperative pain for THA and TKA patients. Instead, the questions were framed broadly for the entire TJA patient group, thereby lacking specificity in addressing the distinct pain management approaches for each procedure. In addition to these limitations, the survey did not explore pain management strategies for patients with chronic pain or those at higher risk for severe postoperative pain, who may require prolonged hospitalization.

This study demonstrates several notable strengths that significantly enhance its reliability and relevance within the field. First, we successfully included all Nordic countries in the survey, ensuring a broad and diverse sample. Our aim was to reach 165 Nordic hospitals performing more than 100 TJAs annually, as well as the hospitals in Iceland that conduct these procedures. We obtained responses from 105 hospitals distributed across a broad region of the Nordic countries. We also received a substantial number of responses from Iceland, despite the country's relatively small size. Additionally, our collaboration with local experts in each country facilitated accurate data collection and provided valuable insights into regional variations. The study also addresses a timely and pressing issue—specifically, day‐case TJAs—making it particularly relevant to ongoing discussions and developments in the research community.

### 4.2 Conclusion

This survey provides valuable insight into current anesthetic and analgesic practices for primary THA and TKA procedures across the Nordic countries. According to the respondents, there appears to be broad consensus on preoperative medication, anesthesia techniques, and multimodal pain management in line with contemporary guidelines. However, variability persists in the use of peripheral nerve blocks and LIA. The widespread implementation of standardized operating procedures reflects a high degree of protocol‐driven care. Although day‐case arthroplasty is increasingly adopted, particularly in Denmark, postoperative pain, motor weakness, and PONV were reported as key barriers to same‐day discharge. Improved and consistent management of PONV, including the routine use of multiple antiemetics, may enhance recovery and support the broader adoption of outpatient joint replacement surgery.

## Author Contributions

M.R., M.T., P.T., and P.U. designed the survey. M.R., A.W., M.I.S., G.G., and S.W. served as national or site coordinators, facilitating survey distribution and assisting with data collection. M.R., M.T., and P.U. analyzed the data. M.T. prepared the figures. All authors critically reviewed the manuscript, approved the final version, and agreed with the findings. All authors read and approved the final manuscript.

## Conflicts of Interest

The authors declare no conflicts of interest.

## Supporting information


**Data S1.** Supporting Information.

## Data Availability

The data that support the findings of this study are available on request from the corresponding author. The data are not publicly available due to privacy or ethical restrictions.
