# Supplementary material for: Anesthesia and Analgesia Methods in Primary Total Hip Arthroplasty and Primary Total Knee Arthroplasty—A Survey of Nordic Anesthesiologists
Source: Acta Anaesthesiol Scand. 2025 Jul 17;69(7):e70091. doi: 10.1111/aas.70091 (PMC12271837; doi:10.1111/aas.70091)
Supplement: Supplementary file 1 — Data S1. Supporting Information. [file AAS-69-0-s001.pdf]

Anesthesia and analgesia methods in primary total hip arthroplasty (THA) and primary total knee arthroplasty (TKA)

Please complete the survey below.

Thank you!

Which Scandinavian country do you work in?	◯ Sweden<br>◯ Norway<br>◯ Denmark<br>◯ Iceland
Where do you work?	◯ University hospital<br>◯ Regional hospital<br>◯ Private hospital
Which hospital do you work at?<br><br>(This question is for study administration only.)	__________________________
What is your work experience?	◯ Resident<br>◯ Consultant 0-5 years<br>◯ Consultant over 5 years
How many patients undergoing primary total hip arthroplasty (THA) or primary total knee arthroplasty (TKA) do you personally treat annually?	◯ 0-40<br>◯ 40-80<br>◯ Over 80
In your unit, is there a standard of practice for anesthesia and analgesia for patients undergoing primary THA or TKA or does each anesthesiologist manage anesthesia and analgesia according to their own preference?	◯ Standard of practice<br>◯ Own preference

Questions about anesthesia and analgesia methods in primary total hip arthroplasty (THA)

Do you use premedication for patients undergoing primary total hip arthroplasty (THA)?
- ◯ Yes
- ◯ No

Which of the following premedication do you use for patients undergoing primary THA if no contraindications occur?
You can choose multiple answers.
- ☐ Benzodiazepine
- ☐ Gabapentinoids
- ☐ NSAIDs
- ☐ Opioids
- ☐ Paracetamol (acetaminophen)
- ☐ Other?

What medication? __________________________________

Do you use intravenous corticosteroids intraoperatively for postoperative pain management for patients undergoing primary THA?
- ◯ Yes, regularly
- ◯ Sometimes
- ◯ No

Which of the following corticosteroids do you use?
- ◯ Betametasone
- ◯ Dexametasone
- ◯ Hydrocortisone
- ◯ Methylprednisolone
- ◯ Other?

What corticosteroid? __________________________________

What is the prevailing anesthesia method in primary THA?
- ◯ Spinal anesthesia
- ◯ General anesthesia
- ◯ Other?

What anesthesia method? __________________________________

Which local anesthetic do you use for spinal anesthesia in primary THA?
You can choose multiple answers.
- ☐ Bupivacaine
- ☐ Mepivacaine
- ☐ Prilocaine
- ☐ Ropivacaine
- ☐ Other?

What local anesthetic? __________________________________

What is the baricity of the local anesthetic of choice in primary THA?
- ◯ Hyperbaric
- ◯ Hypobaric

What is the typical local anesthetic dose (mg) for spinal anesthesia in primary THA?
Provide your answer in milligrams. __________________________________

Do you use intrathecal adjuvants with spinal anesthesia?
- ◯ Yes, regularly
- ◯ Sometimes
- ◯ No

Which of the following intrathecal adjuvants do you use?<br>You can choose multiple answers.	☐ Clonidine<br>☐ Dexmedetomidine<br>☐ Esketamine<br>☐ Opioid<br>☐ Other?
What adjuvant?	__________________________________
How is general anesthesia maintained after induction?<br>You can choose multiple answers.	☐ Balanced volatile anesthesia<br>☐ Total intravenous anesthesia (TIVA)
Which of the following inhalational anesthetic do you use?<br>You can choose multiple answers.	☐ Desflurane<br>☐ Isoflurane<br>☐ Nitrous oxide<br>☐ Sevoflurane<br>☐ Xenon
What is the primary device used for airway management in general anesthesia in primary THA?	◯ LMA<br>◯ Intubation<br>◯ Other?
What airway management device?	__________________________________
Do you use any of the following pain management adjuvants in conjunction with general anesthesia for patients undergoing primary THA?<br>You can choose multiple answers.	☐ Clonidine<br>☐ Dexmedetomidine<br>☐ Esketamine<br>☐ Other?<br>☐ No adjuvants
What pain management adjuvant?	__________________________________
Do you administer ultrasound guided regional anesthesia or any peripheral nerve blocks for pain management in primary THA?	◯ Yes, regularly<br>◯ Sometimes<br>◯ No
Does the choice of anesthesia method (general anesthesia/spinal anesthesia/other) affect whether a peripheral nerve block is provided for patients undergoing primary THA?	◯ Yes<br>◯ No
Which of the following nerve blocks do you administer to patients undergoing primary THA?<br>You can choose multiple answers.	☐ Fascia iliaca compartment block<br>☐ Femoral block<br>☐ Pericapsular Nerve Group (PENG) block<br>☐ Quadratus lumborum block<br>☐ Other?
What nerve block?	__________________________________
Do you use continuous catheter technique for regional anesthesia or periferal nerve block for pain management in primary THA?	◯ Yes, regularly<br>◯ Sometimes<br>◯ No

Which of the following local anesthetic and possible adjuvant do you use for peripheral nerve blocks?<br>You can choose multiple answers.	☐ Bupivacaine<br>☐ Clonidine<br>☐ Corticosteroid<br>☐ Dexmedetomidine<br>☐ Epinephrine<br>☐ Lidocaine<br>☐ Magnesium<br>☐ Mepivacaine<br>☐ Opioid<br>☐ Prilocaine<br>☐ Ropivacaine<br>☐ Other?
What local anesthetic or adjuvant?	____________________________________

Questions about anesthesia and analgesia methods in primary total knee arthroplasty (TKA)

Do you use premedication for patients undergoing primary total knee arthroplasty (TKA)?

- ◯ Yes
- ◯ No

Which of the following premedication do you use for patients undergoing primary TKA if no contraindications occur?
You can choose multiple answers.

- ☐ Benzodiazepine
- ☐ Gabapentinoids
- ☐ NSAIDs
- ☐ Opioids
- ☐ Paracetamol (acetaminophen)
- ☐ Other?

What medication?

____

Do you use intravenous corticosteroids intraoperatively for postoperative pain for patients undergoing primary TKA?

- ◯ Yes, regularly
- ◯ Sometimes
- ◯ No

Which of the following corticosteroids do you use?

- ◯ Betametasone
- ◯ Dexametasone
- ◯ Hydrocortisone
- ◯ Methylprednisolone
- ◯ Other?

What corticosteroid?

____

What is the prevailing anesthesia method in primary TKA?

- ◯ Spinal anesthesia
- ◯ General anesthesia
- ◯ Other?

What anesthesia method?

____

Which local anesthetic do you use for spinal anesthesia in primary TKA?
You can choose multiple answers.

- ☐ Bupivacaine
- ☐ Mepivacaine
- ☐ Prilocaine
- ☐ Ropivacaine
- ☐ Other?

What local anesthetic?

____

What is the baricity of the local anesthetic of choice in primary TKA?

- ◯ Hyperbaric
- ◯ Hypobaric

Do you use any intrathecal adjuvants with spinal anesthesia?

- ◯ Yes, regularly
- ◯ Sometimes
- ◯ No

Which of the following intrathecal adjuvants do you use?
You can choose multiple answers.

- ☐ Clonidine
- ☐ Dexmedetomidine
- ☐ Esketamine
- ☐ Opioid
- ☐ Other?

What adjuvant?	__________________________
What is the typical local anesthetic dose (mg) for spinal anesthesia in primary TKA?<br>Provide your answer in milligrams.	__________________________
How is general anesthesia maintained after induction?<br>You can choose multiple answers.	☐ Balanced volatile anesthesia<br>☐ Total intravenous anesthesia (TIVA)
Which of the following inhalational anesthetic do you use?<br>You can choose multiple answers.	☐ Desflurane<br>☐ Isoflurane<br>☐ Nitrous oxide<br>☐ Sevoflurane<br>☐ Xenon
What is the primary device used for airway management in general anesthesia in primary TKA?	◯ LMA<br>◯ Intubation<br>◯ Other?
What airway management device?	__________________________
Do you use any of the following pain management adjuvants in conjunction with general anesthesia for patients undergoing primary TKA?<br>You can choose multiple answers.	☐ Clonidine<br>☐ Dexmedetomidine<br>☐ Esketamine<br>☐ Other?<br>☐ No adjuvants
What pain management adjuvant?	__________________________
Do you administer ultrasound guided regional anesthesia or any peripheral nerve blocks for pain management in primary TKA?	◯ Yes, regularly<br>◯ Sometimes<br>◯ No
Does the choice of anesthesia method (general anesthesia/spinal anesthesia/other) affect whether a peripheral nerve block is provided for patients undergoing primary TKA?	◯ Yes<br>◯ No
Which of the following nerve blocks do you administer to patients undergoing primary TKA?<br>You can choose multiple answers.	☐ Adductor canal block<br>☐ Femoral block<br>☐ iPACK<br>☐ Other?
What peripheral nerve block?	__________________________
Do you use continuous catheter technique for regional anesthesia or periferal nerve block for pain management in primary TKA?	◯ Yes, regularly<br>◯ Sometimes<br>◯ No

Which of the following local anesthetic and possible adjuvant do you use for peripheral nerve blocks? You can choose multiple answers.

- ☐ Bupivacaine
- ☐ Clonidine
- ☐ Corticosteroid
- ☐ Dexmedetomidine
- ☐ Epinephrine
- ☐ Lidocaine
- ☐ Magnesium
- ☐ Mepivacaine
- ☐ Opioid
- ☐ Prilocaine
- ☐ Ropivacaine
- ☐ Other?

What local anesthetic or adjuvant? ______________________________

Questions about anesthesia and analgesia methods in both primary THA and TKA

Do patients undergoing primary THA or TKA receive preoperative carbohydrate treatment?

◯ Yes
◯ No

When do patients undergoing primary THA or TKA receive preoperative carbohydrate treatment?

◯ In the morning of surgery
◯ The night before and in the morning of surgery
◯ Other?

Please specify.

____

Which sedatives do you use in conjunction with spinal anesthesia for patients undergoing primary THA or TKA?

You can choose multiple answers.

☐ Dexmedetomidine
☐ Midazolam
☐ Remimazolam
☐ Other benzodiazepine
☐ Opioid
☐ Propofol
☐ Other?
☐ No sedatives

What sedative?

____

Is LIA (local infiltration anesthesia) used for patients undergoing primary THA and TKA in your unit?

◯ Yes
◯ No
◯ For THA only
◯ For TKA only

Which medications are included in your LIA in primary THA and TKA?
You can choose multiple answers.

☐ Epinephrine
☐ Corticosteroid
☐ Bupivacaine
☐ Ropivacaine
☐ Lidocaine
☐ NSAID
☐ Other?

What medication?

____

Do patients undergoing primary THA or TKA receive tranexamic acid prophylactically during surgery in your unit?

◯ Yes, regularly
◯ Sometimes
◯ No

Are urinary catheters inserted for patients undergoing primary THA or TKA in your unit?

◯ Yes, regularly
◯ Sometimes
◯ No

In which situations do you insert urinary catheters for patients undergoing primary THA or TKA?

____

Which pain relievers do you regularly use for postoperative pain management for patients undergoing primary THA or TKA if no contraindications occur?
You can choose multiple answers.

☐ Esketamine
☐ Gabapentinoids
☐ NSAIDs
☐ Opioids
☐ Paracetamol (acetaminophen)
☐ Other?

Which pain relievers?	__________________________
Do patients undergoing primary THA or TKA receive routine antiemetic prophylaxis?	◯ Yes<br>◯ No
Which medications do you use for antiemetic prophylaxis?<br>You can choose multiple answers.	☐ 5HT3 antagonist<br>☐ Corticosteroid<br>☐ Droperidol<br>☐ NK1 antagonist

Questions about primary THA and TKA as day surgery

Is primary THA and TKA performed at your unit also as day surgery?
(patient discharge on the same day as surgery)

- ◯ Yes
- ◯ No

What is approximately the portion of day surgery operations of all primary THA and TKA operations in your unit?

- ◯ 0-25%
- ◯ 25-50%
- ◯ 50-75%
- ◯ 75-100%

Does day surgery affect the choice of anesthesia and analgesia?

- ◯ Yes
- ◯ No

Please specify.

____

Does your unit perform day surgery on patients over 75 years old ?

- ◯ Yes
- ◯ No

Does your unit perform day surgery on patients with BMI > 35 kg/m2?

- ◯ Yes
- ◯ No

Does your unit perform day surgery on patients with obstructive sleep apnea with CPAP treatment?

- ◯ Yes
- ◯ No

What are the main reasons for delayed discharge in patients undergoing day surgery?
You can choose multiple anwers.

- ☐ Pain
- ☐ Perioperative blood loss
- ☐ PONV
- ☐ Prolonged motor weakness
- ☐ Urinary retention
- ☐ Other?

What reason?

____

Do you provide postoperative follow-up calls to patients undergoing day surgery?

- ◯ < 48 hours after discharge
- ◯ > 48 hours after discharge
- ◯ No follow-up call
- ◯ Digital follow-up system
- ◯ I don't know

Optional extra question:

Does your unit conduct any active anaesthesiological research on patients undergoing THA/TKA?

- ◯ Yes
- ◯ No
- ◯ I don't know

Kindly add your email address in case we have any additional questions (optional).

We do not share your email address with third parties nor use it for commercial purposes.

____
